# Innate Immune Responses to Wildtype and Attenuated Sheeppox Virus Mediated Through RIG-1 Sensing in PBMC *In-Vitro*

**DOI:** 10.3389/fimmu.2021.666543

**Published:** 2021-06-15

**Authors:** Tesfaye Rufael Chibssa, Richard Thiga Kangethe, Francisco J. Berguido, Tirumala Bharani K. Settypalli, Yang Liu, Reingard Grabherr, Angelika Loitsch, Elena Lucia Sassu, Rudolf Pichler, Giovanni Cattoli, Adama Diallo, Viskam Wijewardana, Charles Euloge Lamien

**Affiliations:** ^1^ Animal Production and Health Laboratory, Joint FAO/IAEA Agricultural and Biotechnology Laboratory, Division of Nuclear Techniques in Food and Agriculture, Department of Nuclear Sciences and Applications, International Atomic Energy Agency, Vienna, Austria; ^2^ Institute of Biotechnology, University of Natural Resources and Life Sciences (BOKU), Vienna, Austria; ^3^ National Animal Health Diagnostic and Investigation Center (NAHDIC), Sebeta, Ethiopia; ^4^ China National Clinical Research Center for Neurological Diseases, Beijing Tiantan Hospital, Capital Medical University, Beijing, China; ^5^ Austrian Agency for Health and Food Safety (AGES), Vienna, Austria; ^6^ Department for Farm Animals and Veterinary Public Health, University Clinic for Swine, University of Veterinary Medicine, Vienna, Austria; ^7^ Laboratoire National d’Elevage et de Recherches Vétérinaires, Institut Sénégalais de Recherches Agricoles (ISRA), Dakar, Sénégal; ^8^ UMR CIRAD INRA, Animal, Santé, Territoires, Risques et Ecosystèmes (ASTRE), Montpellier, France

**Keywords:** gene expression, PRRs, cytokine, RIG-1, LAV, SPPV

## Abstract

Sheeppox (SPP) is a highly contagious disease of small ruminants caused by sheeppox virus (SPPV) and predominantly occurs in Asia and Africa with significant economic losses. SPPV is genetically and immunologically closely related to goatpox virus (GTPV) and lumpy skin disease virus (LSDV), which infect goats and cattle respectively. SPPV live attenuated vaccines (LAVs) are used for vaccination against SPP and goatpox (GTP). Mechanisms related to innate immunity elicited by SPPV are unknown. Although adaptive immunity is responsible for long-term immunity, it is the innate responses that prevent viral invasion and replication before LAVs generate specific long-term protection. We analyzed the relative expression of thirteen selected genes that included pattern recognition receptors (PRRs), Nuclear factor-κβ p65 (NF-κβ), and cytokines to understand better the interaction between SPPV and its host. The transcripts of targeted genes in sheep PBMC incubated with either wild type (WT) or LAV SPPV were analyzed using quantitative PCR. Among PRRs, we observed a significantly higher expression of RIG-1 in PBMC incubated with both WT and LAV, with the former producing the highest expression level. However, there was high inter-individual variability in cytokine transcripts levels among different donors, with the expression of TNFα, IL-15, and IL-10 all significantly higher in both PBMC infected with either WT or LAV compared to control PBMC. Correlation studies revealed a strong significant correlation between RIG-1 and IL-10, between TLR4, TNFα, and NF-κβ, between IL-18 and IL-15, and between NF-κβ and IL-10. There was also a significant negative correlation between RIG-1 and IFNγ, between TLR3 and IL-1 β, and between TLR4 and IL-15 (P< 0.05). This study identified RIG-1 as an important PRR in the signaling pathway of innate immune activation during SPPV infection, possibly through intermediate viral dsRNA. The role of immunomodulatory molecules produced by SPPV capable of inhibiting downstream signaling activation following RIG-1 upregulation is discussed. These findings advance our knowledge of the induction of immune responses by SPPV and will help develop safer and more potent vaccines against SPP and GTP.

## Introduction

Sheeppox (SPP) is a highly contagious viral disease of domestic and wild small ruminants, causing significant economic losses in sheep and goat productivity (1). Sheeppox virus (SPPV), the causative agent of the disease, and two other viruses, goatpox virus (GTPV) and lumpy skin disease virus (LSDV), are the three members of the genus *Capripoxvirus* within the family *Poxviridae* (2). Commonly, SPPV and GTPV cause SPP and GTP in sheep and goats, respectively, while LSDV is restricted to cattle. However, host specificity of SPPV and GTPV is not strict as cross-infection between goat and sheep by those two viruses have been noted (3–5). The two viruses are closely related genetically and immunologically, with some cross-protection observed (6). Given their economic relevance and severity, SPP, GTP, and LSD are listed as notifiable diseases by the world organization for animal health (7). Live attenuated vaccines (LAV) derived from various SPPV strains are used to control SPP and GTP (8–10). Several reports have recognized LAVs as effective vaccines against CaPV infections; however, field data on vaccination suggest some efficacy and safety concerns. There are some vaccination failure cases and adverse reactions (11, 12).

Most SPPV LAVs were developed by serial passages of virulent or low virulent SPPVs in cell culture. Immune responses and host interactions with viruses, including poxviruses, are widely studied *in vitro* using peripheral blood mononuclear cells (PBMC) (13). In addition to fibroblasts, PBMC also includes target cells for SPPV, such as monocytes and macrophages (14). The cytokine expression of PBMC collected from animals vaccinated with SPPV (Romanian vaccine strains), and goatpox virus (Gorgan strain) has previously been studied (15). However, PBMC used in the 2017 study were stimulated with inactivated viruses for the evaluation of immune responses to vaccines using qPCR to measure IL-4 and IFNγ. SPPV infection of sheep PBMC induces an innate antiviral response that leads to the recruitment of antigen-presenting cells which in turn initiate adaptive immunity. Innate immune responses are responsible for recognizing pathogens at the initial encounter through pattern-recognition receptors (PRRs) such as Toll-like receptors (TLRs) and RIG-1-like receptors (RLRs). The recognition of viral pathogens activates a cascade of events leading to the induction of downstream signaling molecules and transcription factors, consequently inducing inflammatory cytokines and chemokines (16). Therefore, the investigation of innate immune responses provides vital information about virus-host interactions. However, little is known on the innate immune response mechanisms elicited by wild-type (WT) and LAV SPPVs. Interrogation of immune responses by directly comparing WT versus LAV SPPVs could provide information on the escape of host immunity by WT. Additionally, any immune markers that LAV induces in the host could also be used for vaccine design.

This study analyzed the innate immune responses to SPPV infections in sheep PBMC. The expression levels of thirteen targeted genes, including pattern recognition receptors (PRRs), Nuclear factor-kβ p65 (NF-κβ), and cytokines in sheep PBMC, were compared, following *in vitro* infections using SPPV WT or LAV viruses.

## Material and Methods

### Viruses

The Wild type isolate SPPV Algeria/93 Djelfa, obtained from the Institut National de la Médecine Vétérinaire, Algiers, Algeria, and Romanian SPPV vaccine, obtained from BioPharma, Morocco, were used for this study. Both viruses were propagated on embryonic skin cell lines from sheep (ESH-L cells) in Hank’s Minimum Essential Medium (MEM) supplemented with 10% fetal calf serum and 1% antibiotics (17). The viral suspensions were titrated (18) and stored at −80°C until further use. All procedures were performed in the Bio-Safety Level-3 laboratory facilities at AGES, Austria.

### PBMC Isolation, CD14- Cell Isolation and Viral Infection

Blood samples were collected from healthy local Austrian sheep *via* jugular vein using heparinized vacutainer tubes and needles. A certified veterinary service collected the samples according to the Austrian Agency for Health and Food Safety (AGES) local guidelines and international guidelines by OIE. PBMC were separated by density-gradient centrifugation using Ficoll-Paque (density, 1.077 g/ml; GE health care, Sweden) and washed twice with RPMI 1640 medium (Gibco, Carlsbad, CA, USA). The resultant pellet was resuspended in complete media (RPMI 1640 media containing 10% FBS and 100 IU/mL penicillin and 100 µg/mL streptomycin) and cell numbers were determined using a hemocytometer. CD14+ monocytes were captured from PBMC using magnetic beads conjugated to a bovine cross-reacting anti-human CD14 monoclonal antibody (human CD14 microbeads, Miltenyi Biotech, Germany) and the CD14- flow-through cells collected as previously described (19). PBMC derived from the donor sheep (n=5) were suspended in complete media and placed in either 24-well plates containing 5 x 10^6^ cells in 1 ml per well (for flow cytometry) or 6-well plates containing 20 x 10^6^ cells in 5 ml per well (for real-time PCR analysis). PBMC were then cultured either with 10µl (flow cytometry) or 100µl (real-time PCR analysis) of WT or LAV strain of SPPV at a concentration of 1x10^6^ TCID50/ml. CD14- cells were also cultured for real-time PCR analysis at 20 x 10^6^ cells in 5 ml with WT or LAV strain of SPPV. Uninfected controls consisting of 10µl or 100µl of PBS added to PBMC or CD14- cell suspensions were also prepared. The plates were incubated at 37°C in a humidified atmosphere containing 5% CO_2_ for 2 days (for real-time PCR analysis) or 4 days (for flow cytometry). The incubation period and the MOI were determined by conducting a series of experiments with variable time points for harvesting and using different MOI.

### RNA Extraction, cDNA Synthesis, and qPCR

After an incubation period of 2 days, PBMC and CD14- cells were harvested, and total RNA extracted from PBMC incubated either with WT or LAV and from control cells using the RNeasy mini kit (Qiagen, Hilden, Germany) following the manufacturer’s instructions with on-column DNAse treatment. The total RNA was resuspended in 30µl of RNase-free water and quantified using a Nanodrop ND-1000 spectrophotometer. Total RNA was converted into cDNA using the SuperScript™ III First-Strand Synthesis System (Invitrogen, USA) as per the manufacturer’s instructions. Approximately one µg of each RNA sample was used with random hexamers according to the manufacturer’s instructions. The resulting cDNA was stored at -20°C until further use. For quantitative analysis, a cDNA dilution of 1: 100 was used as a template for real-time PCR using specific primers presented in **Supplementary Table 1** (20), using GAPDH as a housekeeping gene. Real-time PCR was performed in a total reaction volume of 10 μl containing 2 μl of diluted cDNA as template, 1 μl of forward and reverse primers (**Supplementary Table 1**), 5 μl of iQ SYBR Green Supermix 2X (Bio-Rad, USA), and 1 µl of water. All samples were tested in triplicate. Real-time PCR was performed in a CFX96 real-time PCR Detection System (Bio-Rad, Hercules, USA). The cycling conditions and quantification of targets were performed as previously described (20).

### Quantitative Expression Analysis

The relative fold-change for the thirteen selected genes (**Supplementary Table 1**) in PBMC infected either with WT or LAV compared to PBMC control were analyzed by real-time PCR. These genes were chosen as representative markers from various immune system components, enabling them to interrogate diverse pathways. A panel of specific primers compatible with the same thermal cycling program was used. The specificity of each primer pair was evaluated by melting curve analysis. All amplified PCR products showed distinct and unique Tms that were compatible with the amplification of ovine targets, as previously reported (20). The relative quantities of the target genes were normalized against the internal standard’s relative quantities (GAPDH). Cq values of the amplified templates in sheep PBMC cultured with either WT or LAV were used for the calculation of gene expression. Differences in expression levels were given as fold-change (FC) using the sheep GAPDH gene for normalization and compared with PBMC control.

### Quantification of Viral Growth in PBMC

The quantification of the active virus using cDNA from PBMC and CD14- cells incubated with WT or LAV was carried out with primers and probes targeting the RPO30 gene as previously described (21). Briefly, RNA extracted with on-column DNAse treatment that destroys inoculating SPPV was used as the template for one-step RT-PCR. Absolute quantification of replicating virus was calculated based on the amplification of serially diluted RPO30 plasmid with target copies from 10^8^ to 10^2^ per 2 μL. RNA without reverse transcription was used as a control.

### Surface and Intracellular Cytokine Staining

**Figure 1:**
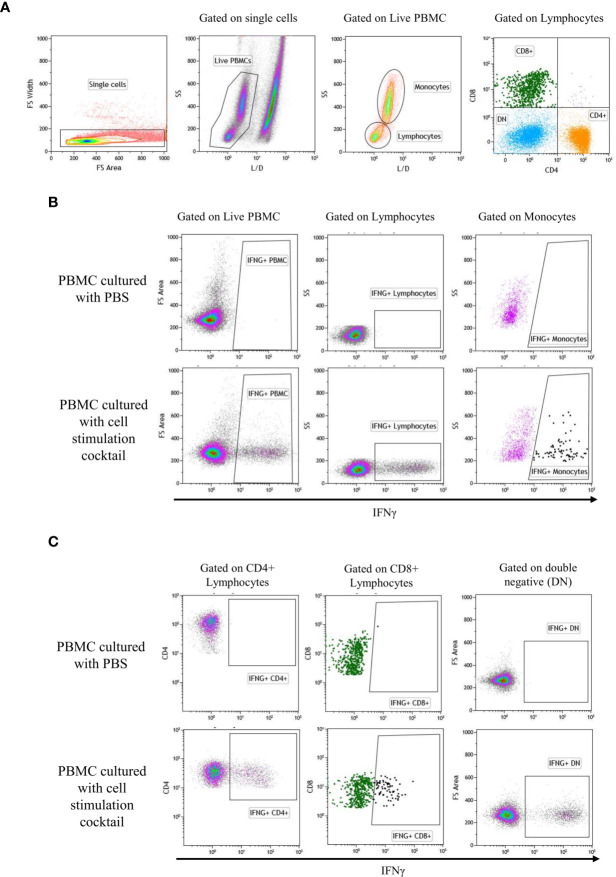
Gate represents gating strategy for Flow cytometry. Analytic gating of flow cytometry showed a representative graph of the change in the mean fluorescence intensity of IFNγ in the PBMC subpopulation. **(A)** Single cells were selected in the forward scatter-height (FSC-HA) versus forwarding scatter-width (FSC-WA) plot, then, live/dead was gated to identify live cells. Next, monocytes and lymphocytes were gated from the live cells on FSC-A versus SSC-A plot. Gating of lymphocyte subset was performed as CD4^+^, CD8^+^, and CD4/CD8 double-negative (DN). **(B)** Expression of IFNγ by PBMC, monocytes, and lymphocytes: IFNγ positive populations were gated using PBMC cultured with PBS (negative control) and PBMC cultured with cell stimulation cocktail (positive control). **(C)** Expression of IFNγ by CD4^+^, CD8^+^, and DN: IFNγ positive populations were gated using PBMC cultured with PBS (negative control) and PBMC cultured with cell stimulation cocktail (positive control).

After 4 days of incubation of PBMC with SPPV (WT or LAV) or PBS, protein transport in cells was blocked by adding BrefeldinA (GolgiStop, BD Biosciences, USA) overnight. Positive control for cytokine production was generated by adding a cell stimulation cocktail (Leukocyte Activation Cocktail with GolgiStop, BD Biosciences) to a third well and incubated overnight. On day 5, PBMC were harvested, washed in PBS, transferred to microcentrifuge tubes, and stained for flow cytometry. Surface staining master mix containing mouse anti-sheep CD4 antibody (clone 44.38: Bio-Rad) and mouse anti-bovine CD8 (clone CC63, cross-reacts with sheep: Bio-Rad) were added to each tube, and samples were incubated at 4°C for 30 min. Following surface staining, dead cell exclusion staining was done with a fixable viability stain (BD Biosciences) before cell fixation. The cells were washed, fixed, and permeabilized with a saponin-based buffer (Fixation/Permeabilization Solution Kit, BD Biosciences) according to the manufacturer’s protocol. Intracellular cytokine staining was done with mouse anti-bovine interferon-gamma antibody (clone CC302, cross-reacts with sheep, Bio-Rad) for 30 min at room temperature in the dark. Cells were washed twice in 1x Perm/Wash buffer and resuspended in FACS buffer (PBS containing 2% FBS and 2mM ethylenediaminetetraacetic acid). Flow cytometry data were acquired using the Gallios flow cytometer (Becton Dickinson Beckman Coulter, USA) and analyzed with Kaluza software (Becton Dickinson, Beckman Coulter). Cell populations were gated by forward and side-light-scatter parameters, as shown in **Figure 1**. Intracellular cytokine expression was calculated as a percentage of the parent population.

### Data Analysis and Presentation

In the present study, gene expression was analyzed using the efficiency-corrected calculation models, based on multiple samples, to estimate the relative changes in the gene expressions (normalized with the GAPDH housekeeping genes) (22). Boxplots of the log_2_ (FC) values and the parent population percentage for flow cytometry data were generated using ggplot2 package in R (23). One sample t-test and two-sample paired t-test were performed in R to compare gene expression differences between the infected and control groups. Infection of PBMC between WT, LAV, PBMC, and CD14- groups were analyzed by fitting a mixed-effects model ANOVA using GraphPad Prism 9. The heatmap.2 function of R gplots package was used to create both expression and correlation heatmaps. The Pearson correlations were computed in R. Hierarchical clustering was performed using the complete linkage method to compute the distance between clusters.

### Bioinformatic Analysis

Based on the previous knowledge that the vaccinia virus E3 and N1L proteins can inhibit the signaling to NF-κβ (24–26), we analyzed their SPPV orthologs in comparison to vaccinia virus and other poxviruses. Sequence alignment of the E3 and the N1L amino acid sequences were done using clustalW implemented in BioEdit version 7.2.6.

The ortholog sequence of N1L from SPPV Djelfa was analyzed using i-Tasser model prediction software (Yang Zhang Laboratory – University of Michigan, Ann Arbor, MI, USA). LOMETS meta-threading software (RCSB Protein Databank) determined the structural similarities based on z-scores. Pymol (Schroedinger, Inc – New York, NY USA) was used to model the structures.

## Results

### Expression Analysis

**Figure 2:**
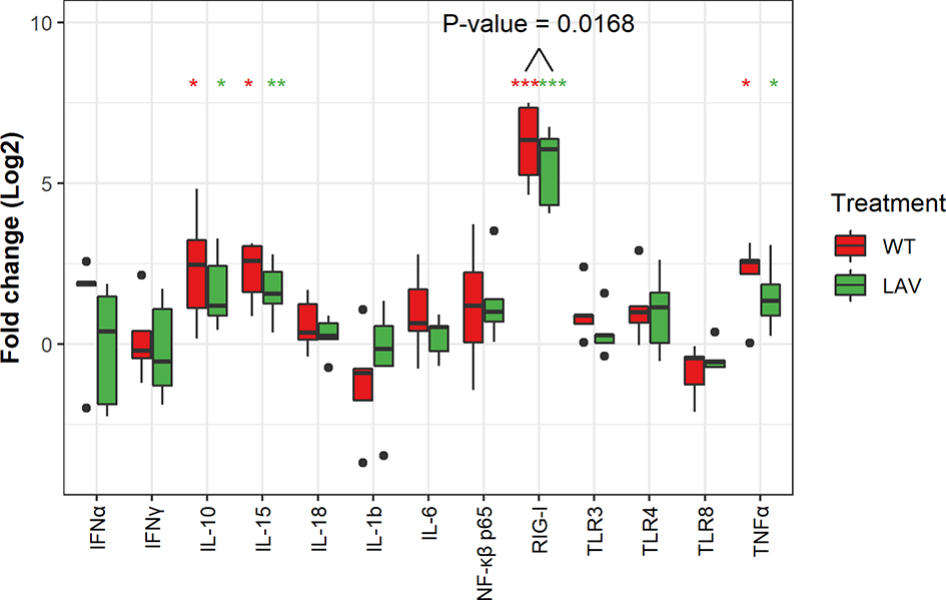
Box plots representing the differential expression of mRNA of thirteen genes. The expression of RIG-1, IL-10, IL-15 and TNFα were significantly upregulated. Note that the expression RIG-1 was significantly higher in WT as compared to LAV. Data represent the Log2 FC of five independent experiments. Significance level was set at P value (*p<0.05, **p<0.001, ***p<0.0001).

The expression level of the transcripts for the PRRs, including TLR3, TLR4, TLR8, and RIG-1, following the infection of sheep PBMC with WT SPPV or LAV SPPV, were converted to log2 fold change and presented in **Figure 2**. At 48 hours post-infection, there was a highly significant expression of RIG-1 in both WT (100-FC) and LAV (60 FC) in infected PBMC cultures (P < 0.001). Moreover, RIG-1 expression in PBMC that were cultured with WT was significantly higher than in those cultured with LAV (P < 0.05). NF-κβ p65 was clearly upregulated in two donors for both LAV and WT; however, the mean fold changes for PBMC infected with either WT (4.3) or LAV (3.7) were not statistically different from that of the control PBMC (P > 0.05; **Figure 2**). The expression level of transcripts for IFNα, IFNγ, TNFα, IL-1β, IL-6, IL-10, IL-15, and IL-18 showed high variability in the cytokine transcripts levels between different donors: 3.1-fold for IL-18 to 28.14-fold for IL- 1β for PBMC treated with LAV; and 4.22-fold for IL-18 to 27.26-fold for IL-1β for those treated with WT. The mean fold changes for TNFα (WT = 5.3, LAV = 3.5), IL-15 (WT = 5.6, LAV = 3.6), and IL-10 (WT = 9.3, LAV = 4.1) were significantly higher in both PBMC infected with either WT or LAV as compared control PBMC (P < 0.05, **Figure 2**). The expression levels of TNFα, IL-15, and IL-10 were higher in PBMC incubated with WT than LAV, though the difference was not significant (P > 0.05). IFNγ, IFNα, IL-6, and IL-18 each presented various patterns of regulation depending on the donor. In general, none of the cytokines were differentially expressed between WT and LAV treatments. These data collectively reveal a greater innate immune gene expression in WT infected sheep PBMC cultures than LAV infections, likely due to increased levels of viral dsRNA within the cytoplasm of infected PBMC. Additionally, inter-individual variability was more pronounced with cytokine transcripts levels.

### Correlation Analysis of an Immune Gene Expression Signature

**Figure 3:**
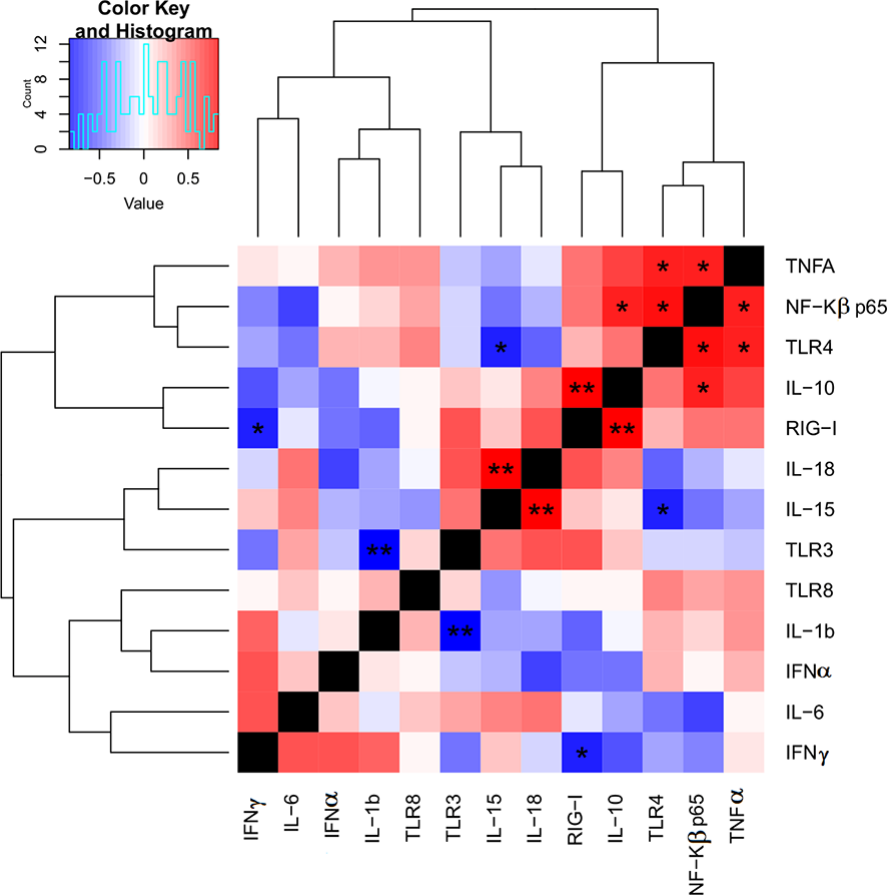
Heat map for correlations of target genes expression FC using qPCR. The correlation heat map describes the combined Pearson correlation coefficient based on the distance between the two gene expression value against all target groups. This shows that the heatmap will cluster together, genes that have positively correlated log2 FC value, P-value; P<0.05*, P<0.01**.

Since many immune markers followed a similar expression pattern between the two (WT and LAV) treatments, we next investigated which markers correlated in their expression. Indeed, heatmap analysis revealed relationships between receptors, transcription factors, and cytokines (**Figure 3**). Our results showed a strong significant correlation between the expression of RIG-1, which recognizes dsRNA, and IL-10 (P< 0.01), and a significant negative correlation between RIG-1 and IFNγ (P< 0.05) in PBMC. TLR3, another PRR that can recognize dsRNA, is moderately correlated with IL-6, IL-15, and IL-18. However, these correlations were not statistically significant. We also observed a highly significant negative correlation between the expression of TLR3 and IL-1 β (P< 0.01) in PBMC. Similarly, there was a significant positive correlation between the expression levels of TLR4, TNFα, and NF-κβ (P< 0.05) and a significant negative correlation between TLR4 and IL-15 (P< 0.05) in PBMC (**Figure 3**). There was a strong and significant positive correlation between the expression level of IL-18 and IL-15 (P< 0.01). The expression of NF-κβ positively and significantly correlated with the expression of IL-10 (P< 0.05).

### Flow Cytometry Analysis of IFNγ in Sheep PBMC Cultured With SPPV

**Figure 4:**
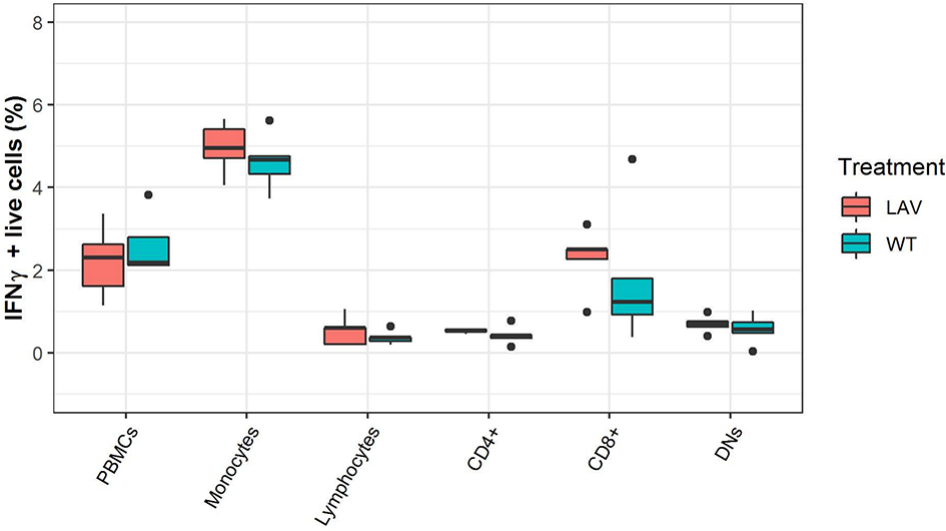
Flow cytometry analysis of the expression of IFNγ in PBMC subpopulations. The box plots represent the percentage of IFNγ expression in healthy sheep PBMC sub-population infected with either WT SPPV or LAV SPPV.

Next, to validate mRNA expressions of immune markers, protein production by individual cell populations within the PBMC were analyzed through flow cytometry. The IFNγ producing cells were identified as a percentage of total live PBMC, monocytes, lymphocytes or CD4+, CD8+, and CD4/CD8 double-negative cells within the lymphocyte population (**Figure 1**). None of the subpopulations had a difference in the percentage of IFNγ producing cells in the identified cell populations between WT or LAV treatments (**Figure 4**). Collectively, the IFNγ production by PBMC detected with flow cytometry was consistent with that determined through quantitative RT-PCR and showed that there was no disparity even among the subpopulation of cells.

### Quantification of Active Virus

**Figure 5:**
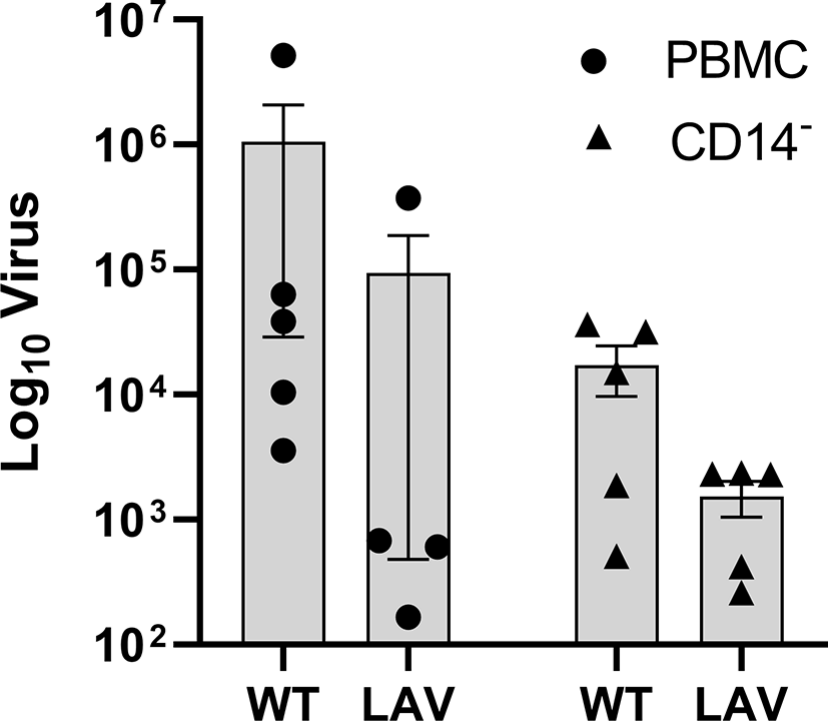
Active viral replication in PBMC and CD14- cells. DNAse treated RNA was used a template for absolute quatification of the RPO30 gene. Viral copies were calculated and the mean (n=5) plotted as box plots with GraphPad Prism 9. Analysis was performed using ANOVA.

The quantification of replicating virus in PBMC incubated with WT virus reveals a higher viral load, close to a log difference, compared to LAV (**Figure 5**). This trend is maintained after removing monocytes from the whole PBMC. More than one log reduction was observed for both WT and LAV in CD14- cells (**Figure 5**). These changes were not statistically significant after analysis was performed. RIG-1 expression was also measured in CD14- cells where an increase was observed when compared to CD14- control cultures (77.5 FC in WT and 77.9 F in LAV; **Supplementary Figure 3**).

### N1L Protein in VACV and SPPV Modeling

To investigate the lack of downstream signal following the activation of RIG-1, including the absence of NF-κβ and type I and type II interferon response, we analyzed the capripoxvirus orthologs of the vaccinia virus N1L and E3 proteins, two known inhibitors of the signaling to NF- κβ. The multiple sequence alignment of SPPV E3 amino acid sequence with other poxviruses’ orthologs showed all essential residues contributing to E3 dsRNA binding (**Supplementary Figure 2**).

In contrast, the N1L of SPPV and other capripoxvirus displayed considerable amino acid sequence divergence to the sequence of vaccinia. Nevertheless, amino acid residues 13, 16, 17, 30, 37, 44, 61 – 78, 89, 92, 98 -102, and 109 – 112 were either identical across all poxviruses or replaced with similar side groups e.g., isoleucine for Leucine in position 13. This prompted the protein quaternary structure modeling compared to the VACV N1L structure (**Supplementary Figure 1**). Sequence alignment of N1L protein from different poxviruses suggests that N1L is highly conserved between sheeppox virus (Djelfa, Roumania), lumpy skin disease virus, and goatpox virus. These substitutions do not affect the quaternary structure of the folded protein (**Supplementary Figure 1**).

## Discussion

We have analyzed the relative expression of thirteen selected genes, encoding for proteins involved in host innate responses to viral infections, following the infection of sheep PBMC with WT SPPV and LAV SPPV to understand better the interaction between SPPV and its host.

Among the mRNA of the pattern recognition receptors (PRRs), we have observed that RIG-1 was highly expressed in PBMC infected with WT and LAV, suggesting that RIG-1 may play an essential role in innate recognition of SPPV. As RIG-1 is a sensor for dsRNA (27), the high expression of this receptor in both WT and LAV suggests that dsRNA intermediates are produced during SPPV infection. Indeed, previous studies showed that some dsDNA viruses, such as poxviruses, that replicate and transcribe their genomes and assemble infectious particles exclusively in the cytoplasm of cells produce dsRNA intermediates (28). The production of IFNγ did not differ between WT and LAV in any of the cell populations when analyzed through flow cytometry, which is consistent with mRNA expression data. However, the highest IFNγ expression was seen in the monocyte cell population. It has already been shown that SPPV can infect monocytes (29). We confirmed this by observing a reduced replication of both WT and LAV in CD14- and no significant difference in the expression of RIG-1 when monocytes are removed from culture. RIG-1 expression was significantly higher in PBMC infected with WT than in those infected with LAV, suggesting that WT infection in PBMC produces more dsRNA intermediates than LAV. Our finding is consistent with the observation that active virus in infected PBMC is one log higher in WT infection than LAV, with this effect likely produced during the infection of monocytes. This suggests that the lower replication of LAV directly results in a lower number of dsRNA intermediates and consequent lower RIG-1 expression when compared to WT virus. This further suggests RIG-1 expression is a direct result of active viral replication yielding dsRNA intermediates. It was interesting to notice that among the tested PRRs, only RIG-1 was significantly positively expressed, suggesting that host sensing of SPPV ds RNA intermediates is mostly through RLRs than TLRs.

There was a high inter-individual variability for cytokines compared to PRRs, though the levels of both cytokines and PPRs appeared to be characteristics of different donors. Indeed, in each donor, the cytokine levels and PRRs levels followed the same trend independently of the SPPV strain used for PBMC infection. Similar inter-individual variabilities in cytokine productions in human PBMC were previously reported and attributed to polymorphisms in genes that control cytokines expression (25, 30). Among the tested cytokines, the expressions of TNFα, IL-15, and IL-10 were all significantly upregulated, suggesting that they may play an essential role in SPPV infections. A similar increase in IL-10 expression following SPPV infection *in vivo* has been reported (31). IL-10 is a critical anti-inflammatory cytokine secreted by monocytes following their infection with pathogens, and increased production of IL-10 following infection by other poxviruses has been previously reported (25, 32). In addition, an IL-10 gene homolog deficient LSDV conferred cross-protective immunity when used as a live attenuated vaccine in both sheep and goats (33). These findings suggest the ability of SPPV to attenuate host immune responses during infection.

Similarly, the increase in TNFα production following poxvirus infection has been reported (25). It was interesting to note the upregulation of inflammatory cytokines IL-15 and TNFα in response to PBMC infection with SPPV. Virus infections result in an inflammatory environment with an increased IL-15 and TNFα and the subsequent recruiting of antigen-presenting cells to the infection site, leading to the induction of an adaptive immune response. However, IL-10 can inhibit those effects. This dichotomy of inflammatory and anti-inflammatory cytokine expressions could also result from the expression of different cytokines from infected and non-infected cells. Therefore, it would be worth looking at cytokine expression after sorting infected and non-infected cells. Interestingly, our data analysis shows a strong positive correlation between the PRR RIG-1 and IL-10, both positively significantly expressed in WT and LAV SPPV infected PBMC.

The strong positive correlation between RIG-1 and IL-10 suggests that the anti-inflammatory properties of IL-10 could mediate immune evasion of SPPV. Anti-inflammatory cytokines such as IL-10 create an inhibitory environment (34). On the other hand, RIG-1 negatively correlated with IFNγ, a necessary cytokine for driving Th1 cell-mediated immunity to control viral infections. Curiously, the activation of RIG-1 did not lead to the subsequent expression of other signaling molecules of the RIG-1 pathway. For instance, our data suggested that the production of NF-κβ and type I and type II interferon response were comparable between treated and control PBMC. The flow cytometry results further supported the absence of specific IFNγ production following PBMC infection with SPPV.

A possible explanation is that SPPV-encoded virokines inhibited RIG-1 downstream signaling leading to a type 1 interferon response. For instance, SPPV possesses VACV orthologous genes encoding for E3, A52, and N1L proteins, all known to interfere with the downstream regulation of RIG-1 (26). Of interest, previous reports have shown that N1L promotes the virulence of VACV (35) through various mechanisms such the inhibition to signaling to NF-κβ (24–26), as well as the IL-1β and TLR4 signaling cascades (25). The sequence alignment of the E3 showed the conservation of essential residues between SPPV and vaccinia virus. The quaternary structure modeling of SPPV N1L, in comparison to VACV, showed that the two proteins display similarities in their structures, suggesting that the SPPV ortholog of N1L protein could play the same function of inhibiting NF-κβ signaling. This signaling is responsible for triggering pro-inflammatory activity in the host, indicating the importance of SPPV N1L as a virulence factor (36).

In conclusion, we have demonstrated that innate immunity is essential as a host defense response against SPPV infections by identifying critical innate mechanisms that follow PBMC exposure to SPPV. The findings will enable us to compensate for deficiencies in our knowledge of cellular mechanisms that activate innate immunity in sheep PBMC infected with SPPV. The data generated will also help develop inactivated vaccines for SPPV and other pox viral diseases in small and large ruminants by incorporating adjuvants that will yield the desired innate immune activation pathway.

## Data Availability Statement

The datasets presented in this study can be found in online repositories. The names of the repository/repositories and accession number(s) can be found in the article/**Supplementary Material**.

## Ethics Statement

Ethical review and approval was not required for the animal study because the blood was collected by a local veterinarian (Viechdoktorei, Tierarztpraxis Leithaprodersdorf OG) and determined clinically healthy. The blood was collected as a part of randomized screening for Maedi-visna and Bluetongue Virus in sheep by the Austrian Agency for Health and Food Safety (AGES).

## Author Contributions

CEL, RTK and VW conceptualized the work, TRC, RTK, RP, ELS and VW carried out the lab experiments, TRC, RTK, YL, VW and CEL analyzed and drafted the manuscript. All authors contributed to the article and approved the submitted version.

## Conflict of Interest

The authors declare that the research was conducted in the absence of any commercial or financial relationships that could be construed as a potential conflict of interest.

## Supplementary Material

Supplementary Figure 1 Ortholog modeling of N1L from SPPV Djelfa. Structural comparison between **(A)** N1L protein of Vaccinia virus (PBD 2I39) and **(B)** SPPV Djelfa ortholog predicted model, **(C)** Overlay of both structures, **(D)** Sequence alignment of N1L protein from different poxviruses.

Supplementary Figure 2 Sequence alignment of E3 protein from different poxviruses.

Supplementary Figure 3 RIG-1 expression in CD14- cells. The expression of RIG-1 was significantly upregulated in CD14- cells. Note that the expression RIG-1 was similar in WT when compared to LAV. Data represent the Log2 FC of five independent experiments.

## References

[1] Carn VM. Control of Capripoxvirus Infections. Vaccine (1993) 11(13):1275–9. 10.1016/0264-410X(93)90094-E 8296478

[2] Tulman ER Afonso CL Lu Z Zsak L Sur J-H Sandybaev NT. The Genomes of Sheeppox and Goatpox Viruses. J Virol (2002) 76(12):6054–61. 10.1128/jvi.76.12.6054-6061.2002 PMC136203 12021338

[3] Diallo A Viljoen GJ. Genus Capripoxvirus. In: Mercer AA Schmidt A Weber O, editors. Poxviruses. Basel, Switzerland: Birkhäuser Advances in Infectious Diseases (2007). Available at: 10.1007/978-3-7643-7557-7_8.

[4] Babiuk S Bowden TR Boyle DB Wallace DB Kitching P. Capripoxviruses: An Emerging Worldwide Threat to Sheep, Goats and Cattle. Transbound Emerg Dis (2008) 55:263–72. 10.1111/j.1865-1682.2008.01043.x 18774991

[5] Wolff J King J Moritz T Pohlmann A Hoffmann D Beer M. Experimental Infection and Genetic Characterization of Two Different Capripox Virus Isolates in Small Ruminants. Viruses (2020) 12(10):1098. 10.3390/v12101098 PMC7600078 32998423

[6] Hamdi J Bamouh Z Jazouli M Boumart Z Tadlaoui KO Fihri OF. Experimental Evaluation of the Cross-Protection Between Sheeppox and Bovine Lumpy Skin Vaccines. Sci Rep (2020) 10(1):8888. 10.1038/s41598-020-65856-7 32483247 PMC7264126

[7] World Organisation for Animal Health (OIE). (2021). Available at: https://www.oie.int/en/disease/sheep-pox-and-goat-pox/ (Accessed June 1, 2021).

[8] Kitching RP. Vaccines for Lumpy Skin Disease, Sheep Pox and Goat Pox. In: Brown F Roth JA, editors. Vaccines for OIE List A and Emerging Animal Diseases. Dev Biol (2003) 114:161–7. 10.1159/issn.1424-6074 14677686

[9] McFadden G. Poxvirus Tropism. Nat Rev Microbiol (2005) 3:201–13. 10.1038/nrmicro1099 PMC4382915 15738948

[10] Tuppurainen ESM Pearson CR Bachanek-Bankowska K Knowles NJ Amareen S Frost L. Characterization of Sheep Pox Virus Vaccine for Cattle Against Lumpy Skin Disease Virus. Antiviral Res (2014) 109(1):1–6. 10.1016/j.antiviral.2014.06.009 24973760 PMC4149609

[11] Norian R Ahangaran NA Vashovi HR Azadmehr A. Evaluation of Humoral and Cell-Mediated Immunity of Two Capripoxvirus Vaccine Strains Against Lumpy Skin Disease Virus. Iran J Virol (2016) 10(4):1–11. 10.21859/isv.10.4.1

[12] Chibssa TR Grabherr R Loitsch A Settypalli TBK Tuppurainen E Nwankpa N. A Gel-Based Pcr Method to Differentiate Sheeppox Virus Field Isolates From Vaccine Strains. Virol J (2018) 15(1):1–7. 10.1186/s12985-018-0969-8 29609650 PMC5879731

[13] Rubins KH Hensley LE Jahrling PB Whitney AR Geisbert TW Huggins JW. The Host Response to Smallpox: Analysis of the Gene Expression Program in Peripheral Blood Cells in a Nonhuman Primate Model. PNAS (2004) 101(42):15190–5. 10.1073/pnas.0405759101 PMC523453 15477590

[14] Gulbahar MY Davis WC Yuksel H Cabalar M. Immunohistochemical Evaluation of Inflammatory Infiltrate in the Skin and Lung of Lambs Naturally Infected With Sheep Pox Virus. Vet Path (2006) 43(1):1–67. 10.1354/vp.43-1-67 16407491

[15] Norian R Ahangaran NA Vashovi HR Azadmehr A. Evaluation of Cell-Mediated Immune Response in PBMCs of Calves Vaccinated by Capri Pox Vaccines Using ELISA and Real-Time RT-PCR. Res Mol Med (2017) 5(2):3–8. 10.29252/rmm.5.2.3

[16] Takeuchi O Akira S. Recognition of Viruses by Innate Immunity. Immunol Rev (2007) 220(1):214–24. 10.1111/j.1600-065X.2007.00562.x 17979849

[17] Lamien CE Lelenta M Goger W Silber R Tuppurainen E Matijevic M. Real Time PCR Method for Simultaneous Detection, Quantitation and Differentiation of Capripoxviruses. J Virol Methods (2011) 171(1):134–40. 10.1016/j.jviromet.2010.10.014 21029751

[18] Reed LJ Muench H. A Simple Method of Estimating Fifty Per Cent Endpoints. Am J Hyg (1938) 27(3):493–7. 10.1093/oxfordjournals.aje.a118408

[19] Kangethe RT Pichler R Chuma F Cattoli G Wijewardana V. Bovine Monocyte Derived Dendritic Cell Based Assay for Measuring Vaccine Immunogenicity In Vitro. Vet Immunol Immunopath (2018) 197:39–48. 10.1016/j.vetimm.2018.01.009 29475505

[20] Sassu EL Kangethe RT Settypalli TBK Chibssa TR Giovanni C Wijewardana V. Development and Evaluation of a Real-Time PCR Panel for the Detection of 20 Immune Markers in Cattle and Sheep. Vet Immunol Immunopath (2020) 227:110092. 10.1016/j.vetimm.2020.110092 32673891

[21] Settypalli TBK Lamien CE Spergser J Lelenta M Wade A Gelaye E. One-Step Multiplex RT-Qpcr Assay for the Detection of *Peste des petits ruminants virus*, *Capripoxvirus*, *Pasteurella multocida* and *Mycoplasma capricolum subspecies (ssp.) capripneumoniae* . PloS One (2016) 11(4):e0153688. 10.1371/journal.pone.0153688 27123588 PMC4849753

[22] Pfaffl MW. A New Mathematical Model for Relative Quantification in Real-Time RT-PCR. Nucleic Acids Res (2001) 29(9):e45. 10.1093/nar/29.9.e45 11328886 PMC55695

[23] Wickham H. Ggplot2: Elegant Graphics for Data Analysis. Cham, Switzerland: Springer (2016). Available at: 10.1007/978-3-319-24277-4.

[24] DiPerna G Stack J Bowie AG Boyd A Kotwal G Zhang Z. Poxvirus Protein N1L Targets the I-Kb Kinase Complex, Inhibits Signaling to NF-κB by the Tumor Necrosis Factor Superfamily of Receptors, and Inhibits NF-κB and IRF3 Signaling by Toll-Like Receptors. J Biol Chem (2004) 279(35):36570–78. 10.1074/jbc.M400567200 15215253

[25] Zhang Z Abrahams MR Hunt LA Suttles J Marshall W Lahiri DK. The Vaccinia Virus N1L Protein Influences Cytokine Secretion in Vitro After Infection. Ann N Y Acad Sci (2005) 1056:69–86. 10.1196/annals.1352.005 16387678

[26] Smith GL Talbot-Cooper C Lu Y. How Does Vaccinia Virus Interfere With Interferon? Advs Virus Res (2008) 100:355–78. 10.1016/bs.aivir.2018.01.003 29551142

[27] Dixit E Kagan JC. Intracellular Pathogen Detection by RIG-I-Like Receptors. Advs Immunol (2013) 117:99–125. 10.1016/B978-0-12-410524-9.00004-9 PMC3947775 23611287

[28] Moss B. Poxvirus DNA Replication. Cold Spring Harb Perspect Biol (2013) 5:a010199. 10.1101/cshperspect.a010199 23838441 PMC3753712

[29] Embury-Hyatt C Babiuk S Manning L Ganske S Bowden TR Boyle DB. Pathology and Viral Antigen Distribution Following Experimental Infection of Sheep and Goats With Capripoxvirus. J Comp Pathol (2012) 146(2-3):106–15. 10.1016/j.jcpa.2011.12.001 PMC9528194 22297076

[30] Yaqoob P Newsholme EA Calder PC. Comparison of Cytokine Production in Cultures of Whole Human Blood and Purified Mononuclear Cells. Cytokine (1999) 11(8):600–5. 10.1006/cyto.1998.0471 10433807

[31] Abu-El-Saad AAS Abdel-Moneim AS. Modulation of Macrophage Functions by Sheeppox Virus Provides Clues to Understand Interaction of the Virus With Host Immune System. Virol J (2005) 2:22. 10.1186/1743-422X-2-22 15784144 PMC1079960

[32] Wong PS Sutejo R Chen H Ng SH Sugrue RJ Tan BH. A System Based-Approach to Examine Cytokine Response in Poxvirus-Infected Macrophages. Viruses (2018) 10(12):692. 10.3390/v10120692 PMC6316232 30563103

[33] Boshra H Truong T Nfon C Bowden TR Gerdts V Tikoo S. A Lumpy Skin Disease Virus Deficient of an IL-10 Gene Homologue Provides Protective Immunity Against Virulent Capripoxvirus Challenge in Sheep and Goats. Antiviral Res (2018) 123:39–49. 10.1016/j.antiviral.2015.08.016 26341190

[34] Subramanian Iyer S Cheng G. Role of Interleukin 10 Transcriptional Regulation in Inflammation and Autoimmune Disease. Crit Rev Immunol (2012) 32(1):23–63. 10.1615/CritRevImmunol.v32.i1.30 22428854 PMC3410706

[35] Bartlett N Symons JA Tscharke DC Smith GL. The Vaccinia Virus N1L Protein Is an Intracellular Homodimer That Promotes Virulence. J Gen Virol (2002) 83(8):1965–76. 10.1099/0022-1317-83-8-1965 12124460

[36] Maluquer de Motes C Cooray S Ren H Almeida GM McGourty K Bahar MW. Inhibition of Apoptosis and NF-κβ Activation by Vaccinia Protein N1 Occur Via Distinct Binding Surfaces and Make Different Contributions to Virulence. PloS Pathog (2011) 7(12):e1002430. 10.1371/journal.ppat.1002430 22194685 PMC3240604

