# Supplementary figures and images for: Innate Immune Responses to Wildtype and Attenuated Sheeppox Virus Mediated Through RIG-1 Sensing in PBMC In-Vitro
Source: Front Immunol. 2021 Jun 15;12:666543. doi: 10.3389/fimmu.2021.666543 (PMC8240667; doi:10.3389/fimmu.2021.666543)

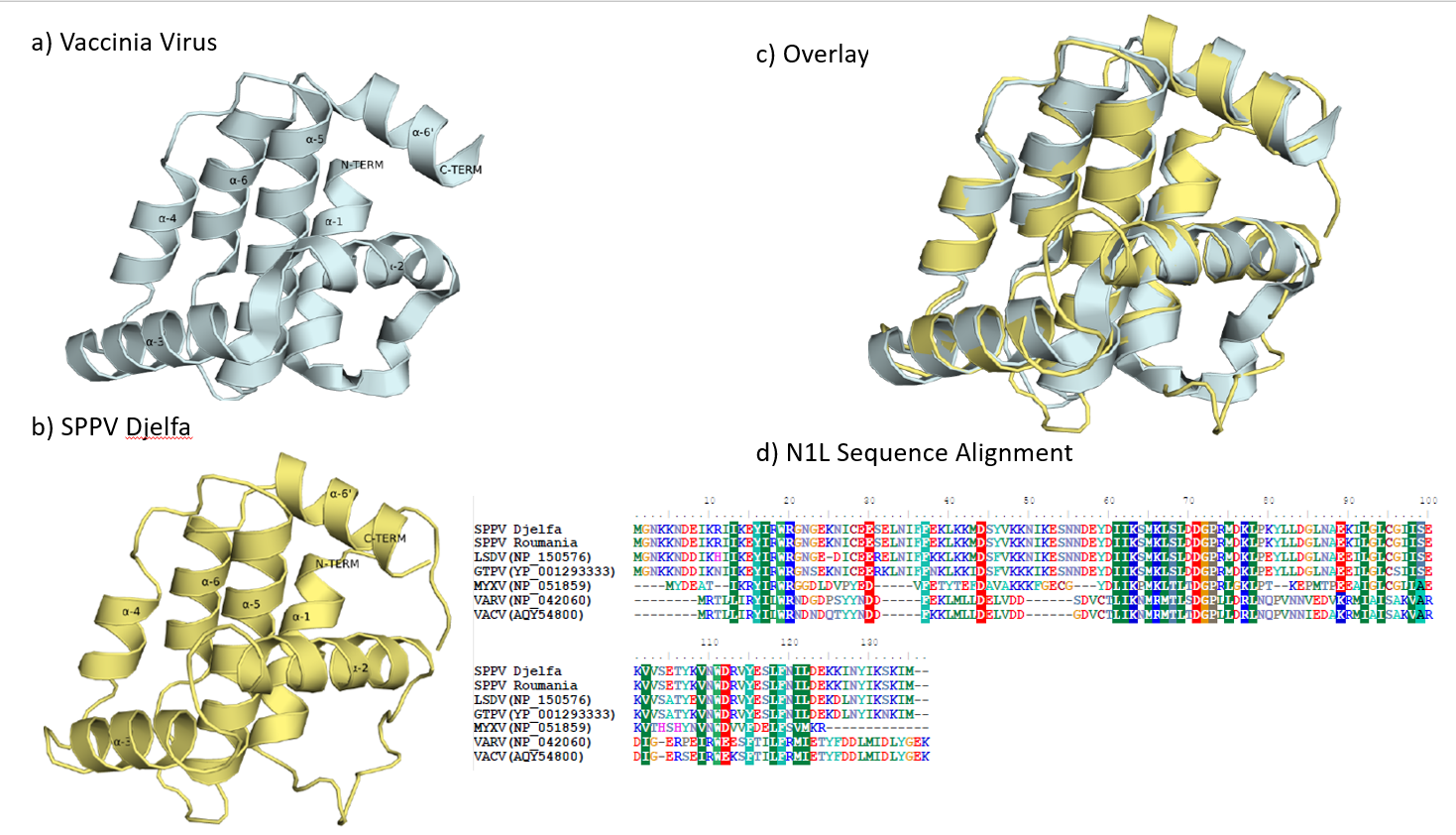

Supplement: Supplementary Figure 1 — Ortholog modeling of N1L from SPPV Djelfa. Structural comparison between (A) N1L protein of Vaccinia virus (PBD 2I39) and (B) SPPV Djelfa ortholog predicted model, (C) Overlay of both structures, (D) Sequence alignment of N1L protein from different poxviruses. [file Image_1.tif]

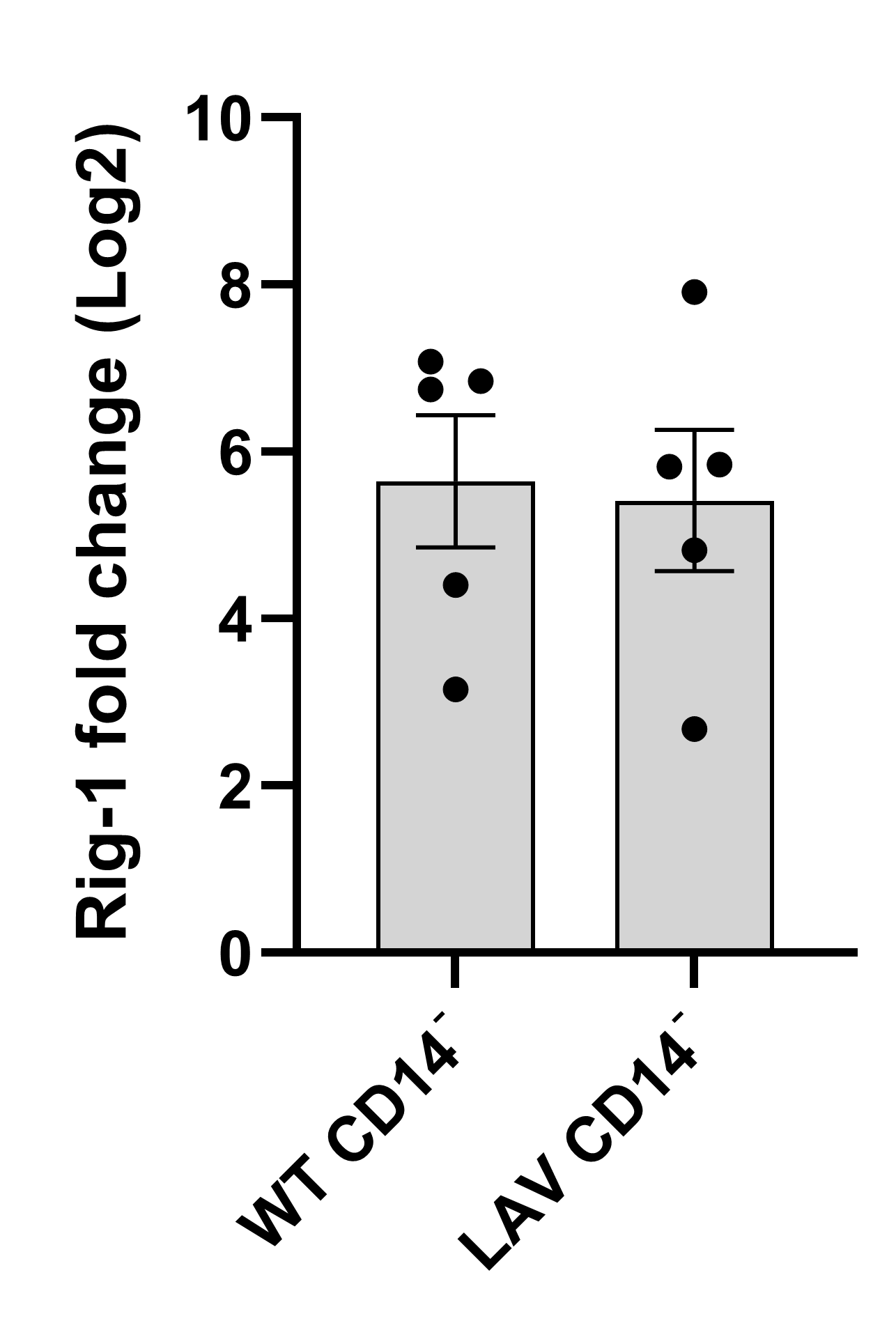

Supplement: Supplementary Figure 2 — Sequence alignment of E3 protein from different poxviruses. [file Image_2.tif]
