# Supplementary material for: Innate Immune Responses to Wildtype and Attenuated Sheeppox Virus Mediated Through RIG-1 Sensing in PBMC In-Vitro
Source: Front Immunol. 2021 Jun 15;12:666543. doi: 10.3389/fimmu.2021.666543 (PMC8240667; doi:10.3389/fimmu.2021.666543)
Supplement: Supplementary file 4 [file Table_1.docx]

**Supplementary Table 1. Primer sequences for real-time PCR amplification of sheep target genes (Sassu et al., 2020)**

| **Gene**  **family** | **Target gene** | **Primer sequences (5’-3’)** | **Primer conc. pmol/µl** | **Amplicon size (bp)** | **Tm (^o^C)** |
| --- | --- | --- | --- | --- | --- |
| **Interleukins** | TNFα | F-CCAGAGGGAAGAGCAGTCC | 1.25 | 112 | 84 |
|  |  | R-GGCTACAACGTGGGCTACC |  |  |  |
|  | IFNγ | F-CAGAGCCAAATTGTCTCCTTC | 1.25 | 168 | 81 |
|  |  | R-ATCCACCGGAATTTGAATCAG |  |  |  |
|  | IFNɑ | F-GTGAGGAAATACTTCCACAGAGTCACT | 1.25 | 107 | 81.50 |
|  |  | R-TGARGAAGAGAAGGCTCTCATGA |  |  |  |
|  | IL-1β | F-ATTGCCCAGGTTTCTGAAACA | 2.5 | 78 | 79 |
|  |  | R-CTCGTCACTGTAGTAAGCCATCATTT |  |  |  |
|  | IL-6 | F-CCGCTTCACAAGCGCCTTC | 2.5 | 248 | 82.50 |
|  |  | R-CCAGTGTCTCCTTGCTGCTT |  |  |  |
|  | IL-10 | F-CTTTAAGGGTTACCTGGGTTGC | 1.25 | 262 | 87.50 |
|  |  | R-CTCACTCATGGCTTTGTAGACAC |  |  |  |
|  | IL-15 | F-TTCATGTCTTCATTTTGGGCTGT | 2.5 | 181 | 80.50 |
|  |  | R-AAGCACTGCATCGCTGTTAC |  |  |  |
|  | IL-18 | F-GCTGCCGTCTTCTGTAAGGA | 1.25 | 191 | 81.50 |
|  |  | R-TCCAGGTCTTCATCATTTTCAGC |  |  |  |
| **PRRs** | TLR3 | F-CTCCCCAATGGAGGACGAAG | 2.5 | 195 | 80.50 |
|  |  | R-GCTTGCTGAACTGCATGGTG |  |  |  |
|  | TLR4 | F-TCCCCGACAACATCCCCATA | 1.25 | 224 | 83 |
|  |  | R-AAAGGCTCCCCAGGCTAAAC |  |  |  |
|  | TLR8 | F-CCCTGACCCAACTTCGCTAC | 2.5 | 137 | 82.50 |
|  |  | R-TAAGGAGGGCATTTCGTCCA |  |  |  |
|  | RIG-1 | F-GCCTTAAAGAACTGGATTGA | 1.25 | 95 | 82.50 |
|  |  | R-ATACCCATTGTCTGATTTGTT |  |  |  |
| **NF-κB** | NF-κβ p65 | F-CGGGGACTACGACCTGAATG | 2.5 | 250 | 88.50 |
|  |  | R-GCCTGGTCCCGTGAAATACA |  |  |  |
| **Control** | GAPDH | F-CCTGGAGAAACCTGCCAAGT | 2.5 | 214 | 85.50 |
|  |  | R-GCCAAATTCATTGTCGTACCA |  |  |  |

RIG-1 = retinoic acid-inducible gene I; TLR3 = Toll-like receptor 3; TLR4 = Toll-like receptor 4; TLR8 = Toll-like receptor 8; IL-18 = Interleukin 18;  IL-15 = Interleukin 15; IL-10 = Interleukin 10; IL-6 = Interleukin 6; IFNγ = Interferon Gamma; IFNɑ = Interferon alpha, TNFα = Tumor Necrosis Factor Alpha; NF-κβ p65 = nuclear factor kappa-light-chain-enhancer of activated B cells; IL-1β = Interleukin 1 beta GAPDH = Glyceraldehyde 3-phosphate dehydrogenase; Tm = melting temperature
