# Supplementary material for: Innate Immune Responses to Wildtype and Attenuated Sheeppox Virus Mediated Through RIG-1 Sensing in PBMC In-Vitro
Source: Front Immunol. 2021 Jun 15;12:666543. doi: 10.3389/fimmu.2021.666543 (PMC8240667; doi:10.3389/fimmu.2021.666543)
Supplement: Supplementary Figure 3 — RIG-1 expression in CD14- cells. The expression of RIG-1 was significantly upregulated in CD14- cells. Note that the expression RIG-1 was similar in WT when compared to LAV. Data represent the Log2 FC of five independent experiments. [file DataSheet_1.pdf]

```
                              10        20        30        40        50        60        70        80        90       100
                     ....|....|....|....|....|....|....|....|....|....|....|....|....|....|....|....|....|....|....|....|
SPPV Djelfa          ---MYS-CDEVDSYELVKKMVNNLSESESITAIEISKKLNIEKSNVNKQLYKLHNDGFIFMIRSNPPKWFKKNGIDNDDNENNNTKK------------L
SPPV Roumania        ---MYS-CDEVDSYELVKKMVNNLSESESITAIEISKKLNIEKSNVNKQLYKLHNDGFIFMIRSNPPKWFKKNVIDNDDNENNNTKK------------L
LSDV(NP_150468)      ---MYS-CDEVDSYELVKKMVNNLSESEFITAIEISRKLNIEKSNVNKQLYKLHNDGFIFMIRSNPPKWFKKNSIDNDDNENNDTKK------------L
GTPV(YP_001293225)   ---MYS-FDEVDSYELVKKMVNNLSESEFITAIEISKKLNIEKSNVNKQLYKLHNDGFIFMIRSNPPKWFKKNGIDNNDNDNNDTKK------------L
TPV(ABQ43508)        MDLLSCTVNDAEIFSLVKKEVLSLNTNDYTTAISLSNKLKIDKKKINQQLYKLKKEDTVKMVPSNPPKWFKNYNGEH----DSKLEQKHHI--------P
SPV(NP_570192)       ---MCSDISNEDVYSLVKQEVDSLPVGNFITAVEISKKIEKEKSSINRQLYALYQQGYIDMVPACPPKWYKRN-QDNMNNESIEIHN------------P
YMTV(NP_938290)      MDSPGC-ENDVKTFSLVKNEVMMLNVDEYTTSIDISNKLKINKKKINKQLYKLQKEGVLKMVPSNPPKWFKNCNCEDSENNDNKLESRDHV--------P
CPXV(SNB53872)       --MSKIYIDERSDAEIVCEAIKNIGREG-ATAVQLTKQLNMEKREVNKALYDLQRSAMVYSSDDTPPRWFTTMDADTQPTDSDADDIIDDVSREKSMRDD
VACV(AAA48040)E3L    --MSKIYIDERSDAEIVCAAIKNIGIEG-ATAAQLTRQLNMEKREVNKALYDLQRSAMVYSSDDIPPRWFMTTEADKPDADAMADVIIDDVSREKSMRED
MYXV(NP_051743)      ------------------------------------------MDPINTLWHNALSSGNAAVSETKP----------------------------------

                             110       120       130       140       150       160       170       180       190
                     ....|....|....|....|....|....|....|....|....|....|....|....|....|....|....|....|....|....|...
SPPV Djelfa          NKSFSDTIPYYKIVLWKEKNPCSAINEYCQFTSRDWYINISSCGNGRKPMFLASVIISGIKFFPEIGNTKKEAKQKSTKRTIDFLINTSIIKF
SPPV Roumania        NKSFSDTIPYYKIVLWKEKNPCSAINEYCQFTSRDWYINISSCGNGRKPMFLASVIISGIKFFPEIGNTKKEAKQKSTKRTIDFLINTSIIKF
LSDV(NP_150468)      NKSFSDTIPYYKIVLWKEKNPCSAINEYCQFTSRDWYINISSCGNGRKPMFLASVIISGIKFFPEIGNTKKEAKQKSTKRTIDFLINTSIIKF
GTPV(YP_001293225)   NKSFSDTIPYYKIVLWKEKNPCSAINEYCQFTSRDWYINISSCGNGRKPMFLASVIISGIKFFPEIGNTKKEAKQKSTKRTIDFLINTSIIKF
TPV(ABQ43508)        NHIFSDTVPYKKIINWKDKNPCIVLNEYCQFTCRDWSIDITTSGKSHCPMFTATVIISGIKFKPAIGNTKREAKYNASKITMDEILDSVIIKF
SPV(NP_570192)       DHMFSDTIPYTKIIEWKNKNPITVLNEYCQITQRDWIIDIISSGQSHCPIFTASITVSGIKCKTGKGSTKKEAKQIAARETMNFILNKTIIKF
YMTV(NP_938290)      HHIFKDEIPYKKIISWKDKTPCTVINEYCQLTSRDWSIEVTTAGESHCPIFTASVIISGIKFKPEIGNTKKEAKHKASKITMEEILKSSIVKF
CPXV(SNB53872)       HKSFDDVIPAKKIIDWKGANPVTVINEYCQITKRDWSFRIESVGPSNSPTFYACVDIDGRVFDKADGKSKRDAKNNAAKLAVDKLLGYVIIRF
VACV(AAA48040)E3L    HKSFDDVIPAKKIIDWKDANPVTIINEYCQITKRDWSFRIESVGPSNSPTFYACVDIDGRVFDKADGKSKRDAKNNAAKLAVDKLLGYVIIRF
MYXV(NP_051743)      --IFGDTIKYEKIVSWQTKNPCTVLNEYCQITFREWSINVTRAGQSHSPTFTAVVTVSGYSFKSATGSNKKEARKNAAKEAMDVILKHVVIKF
```
